# Meeting of the Georgia Medical Association

**Published:** 1897-05

**Authors:** 


### Editorial.

---

### MEETING OF THE GEORGIA MEDICAL ASSOCIATION.

The State Medical Association convened at Macon, April 21, and closed April 23.

The meeting was in every way successful. The weather was all that could be desired, the attendance was large and the papers presented were of a high order and freely and interestingly discussed. The local committee of arrangement, under the leadership of Dr. Howard J. Williams, were undefatigable in their efforts to make the meeting sociably enjoyable to the visitors, and the success of their endeavors was manifest in the expressions of commendation on the part of the visiting members.

The association was hospitably and elegantly entertained at the receptions given by Dr. K. P. Moore and the Cherokee Club on Tuesday. On Thursday there was a fine barbecue served at Ocmulgee Park with the delightful accompaniment of music.

In the afternoon before the barbecue Dr. Samuel Lloyd, of New York, gave an interesting demonstration of the Roentgen ray to a large audience.

On Friday, the distinguished Virginian, Dr. Hunter McGuire, of Richmond, read an instructive paper on appendicitis, the text of which will be found elsewhere in this number. It is an inexpressible pleasure for the Georgia Association to entertain Dr. McGuire, who was so closely associated with the cause which, though dead, is still held in reverence by every true son of the South. His paper was freely and intelligently discussed.

The officers for the ensuing year were elected, with J. B. Morgan, of Augusta, President; L. G. Hardman, of Harmony Grove, First Vice-President; J. Lawton Hiers, of Savannah, Second Vice-President, and Chas. Hicks, Censor, succeeding himself.

Dr. Morgan is entirely deserving of the honor conferred upon him. His clear and deliberate, dignified manner, and ready grasp of the strong points of a subject, render him admirably fitted for the part of presiding officer. Dr. Hardman, who has already achieved an enviable reputation as a surgeon, and as an able and forceful speaker in medical assemblies, will reflect great credit upon the Association, and was appropriately chosen. Dr. Hiers will fill with credit the office of Second Vice-President, and the genial Hicks will very properly continue one of the solid members of the Board of Censors. Brunswick was chosen as the next place of meeting.

The Macon meeting will always be recalled with pleasure. The rapid and interesting trip on the Central, the cordiality and hospitality of the local physicians, the instructive meetings, and seeing again familiar faces, all served to make the occasion one of enjoyment and profit.

